# What do they TEL(L)? A systematic analysis of master programs in technology-enhanced learning

**DOI:** 10.1186/s41239-021-00305-7

**Published:** 2022-01-06

**Authors:** Mikhail Fominykh, Joshua Weidlich, Marco Kalz, Ingunn Dahler Hybertsen

**Affiliations:** 1 Norwegian University of Science and Technology, Trondheim, Norway; 2 Heidelberg University of Education, Heidelberg, Germany

**Keywords:** Technology-enhanced learning, Study program analysis, Curriculum analysis, Hierarchical cluster analysis

## Abstract

This article contributes to the debate on the growing number of interdisciplinary study programs in learning and technology, and aims to understand the diversity of programs as well as curricula structure in an international landscape. Scientific fields share their knowledge and recruit young researchers by offering discipline-specific study programs. Thus, study programs are a reflection of the fields they represent. As technology-enhanced learning is considered to be particularly interdisciplinary and heterogenous, it is important to better understand the landscape of study programs that represents the field. This article presents an analysis of master programs in technology-enhanced learning. A systematic review and analysis of master programs offered in English has been conducted and further used as input for hierarchical cluster analysis. The study identified general characteristics, curricula structure, and organization of topics of these programs. Hierarchical cluster analysis and qualitative content analysis helped us to identify the major types of curricular structures and typical topics covered by the courses. Results show that most study programs rely on interdisciplinary subjects in technology-enhanced learning with a considerable number of subjects from education, learning and psychology. Subjects related to technology, information and computer science appear in such programs less frequently.

## Introduction

The field of technology-enhanced learning (TEL) is a relatively young research domain. The term was coined in the context of working groups initiated by the European Commission in 2000. It is now broadly used to designate “a field of research aiming at improving learning by integrating current technologies and designing innovative ones” (Bourdeau & Balacheff, 2014). Kirkwood and Price (2014) state that the term has been used since around 2005 to describe a research focus concentrating on the impact of technology on learning. Similar to its broader and longer-established counterpart, educational technology, the research focus of TEL has led to interdisciplinary perspectives integrating theories and methods from education, psychology, and computer science (Conole et al., 2010; Kalz & Specht, 2014; Scanlon & Taylor, 2016). Despite justified criticism around the concept (Bayne, 2015), the term has not only been used to describe a research domain, it has also been the foundation for a number of higher education study programs (Orey, 2017). This landscape has been characterized as continuously changing (Dennen & Spector, 2007; Spector, 2015). At the same time, there is a relative lack of research that has systematically reviewed topics, themes, and trends of curricula in study programs on TEL.

This lack of research is concerning, because knowledge of the international landscape of TEL education can provide us with useful information. For example, it can provide an understanding of which topics, themes, and trends in research end up being translated into higher education curricula. It may allow us to understand the current state of qualification, that is, which skills and competences can be expected from recently graduated TEL students entering the job market or academia. It can also provide a picture of what the field actually finds valuable, foundational, and/or tangential. In addition, a systematic overview on existing TEL programs could identify typical structures, similarities and differences between programs.

Furthermore, a comprehensive study of TEL programs could provide theoretical and methodological implications contributing to better understanding of interdisciplinary fields and to the development of new study programs. The research field of TEL is often being referred to as interdisciplinary. TEL study programs can be seen as interdisciplinary when they include courses where instructors from different disciplines work together to illuminate a shared topic. In contrast from multidisciplinary programs, interdisciplinary designs have a distinct goal to integrate different disciplines and illuminate different sides of the same topic (Ivanitskaya et al., 2002; Troelsen et al., 2015; Yang, 2009).

As related work, Sommerhof et al. (2018) conducted a document analysis of 75 graduate programs in the learning sciences, providing an overview of concepts and methods that make up the landscape of learning science education. Based on the homogeneity of programs with respect to certain core characteristics (e.g., a focus on design-based research), they went on to draw broader conclusions as to the nature of the learning science community, identifying it as a true community of practice. While certainly similar to the present study, both in scope and rationale, the analysis of Sommerhof et al. (2018) was specific to the learning sciences. Despite being a discipline with many shared interests with TEL, foundational differences in focus (e.g., the importance of technology) do not allow us to readily apply these findings to TEL.

As related work that is more specific to TEL, Orey (2017) identified, through web search and collecting self-reports of institutions, a large array of graduate programs in the domains of learning, design, and technology. Intended as a resource rather than scholarly research, this list mainly provided information as to the structure, administration, and prestige-metrics (i.e. awards) of these programs. Thus, a central drawback was that no further analysis had been provided. An updated overview of TEL master programs may similarly function as an important resource, with the possibility of providing orientation for prospective students interested in TEL. Similarly, higher education institutions looking to add a TEL program to their offerings may benefit from knowing what is “out there”. This is especially useful information after the current ad-hoc shift to online-learning during the Covid-19 pandemic. Going beyond this, a content-based analysis of similarities and differences of TEL programs also allows us to assess if there are groups of programs with distinct characteristics. Considering the self-image of the TEL research field being characterized by a high degree of interdisciplinarity, it may be interesting to investigate how this is represented in TEL education.

Providing a different perspective on TEL education, Hartley et al. (2010) delineated 13 curricular key themes as well as a framework encompassing five competency domains. These, the authors argue, are the very basic building blocks of what a TEL program should encompass and what a graduate student exiting these programs should have been made familiar with. Thus, instead of assessing what is there, these authors outlined what should be there. However, as has been noted (Spector, 2015), it appears that currently no program has fully embraced these standards. Many higher education institutions aim to incorporate the first three competencies (knowledge, process, and application domain) into their core curricula and outsource the following two competencies (personal/social and innovative/creative domain) to projects and internships (Mayrberger & Kumar, 2014).

Arrington and Darabi (2018) derived quality indicators for TEL programs by asking students and faculty. They found course topic variety, relevancy and currency to the field, as well as curriculum quality to be among the most frequently mentioned quality themes. Both students and faculty agreed on the importance of these indicators while diverging on others. The uniform attention paid to these themes further highlights the significance of an analysis of TEL programs, as topic variety, relevance to practice, and curriculum quality all relate to content of programs, that is, what the designers of the program choose to include as representative for TEL.

Last but not least, Pammer-Schindler et al. (2020) analyzed doctoral training in the field of TEL. They surveyed a sample of education programs in Europe in which doctoral students working on TEL topics are enrolled. The findings indicate that most doctoral schools are associated with a single discipline and offer methodological rather than content-specific modules. TEL-specific content is provided only in exceptional cases, creating a potentially isolating gap between master-level education and scientific conferences.

Based on the identified gaps in the literature, the objective of the current study was to get an international overview of master’s study programs that focus on TEL. Thus, the overarching research question for the study was:

### RQ

What is the current landscape of TEL master programs?

In order to operationalize this very broad question, more specific research questions were derived based on the above argumentation:

### RQ1

What are the characteristics of TEL master programs?

### RQ2

How distinct are TEL master programs in terms of their curricula?

### RQ3

How are course topics organized in TEL master programs?

In order to address these research questions, a systematic and comprehensive search of program databases was conducted, yielding a corpus of metadata about TEL programs that fit our criteria. In the second phase, a hierarchical cluster analysis of the data was conducted. The following chapter describes the systematic search process, the search protocol, our inclusion criteria, as well as the steps towards a cluster analysis of these data extracted about these programs. Finally, the results of the analysis will be presented before we discuss the findings based on our research questions.

## Method

The study was conducted in two phases. In the initial phase, study programs were identified via a systematic search in public online portals. In the second phase, the data were used for hierarchical cluster analysis. We describe both phases in detail below.

### Phase 1: Systematic analysis of programs

#### Sample definition

Before conducting our systematic search, we defined inclusion and exclusion criteria. Since our analysis aimed to provide an overview with international scope, we primarily aimed at study programs offered in English (first inclusion criterion). We did not however focus on international master programs exclusively.

The second inclusion criterion was the status of the Higher Education institution as an accredited institution as well as the level of the program, either on Master’s level (1–2 years) or programs on the bachelor’s plus Master’s level (4–5 years). By defining these inclusion criteria, we excluded shorter programs and certificate courses.

The following inclusion criteria are related to the curricula of the programs. We defined three TEL-relevant domains of subjects:

- Subjects related to education, learning and psychology
- Subjects related to technology, information and computer science
- Interdisciplinary TEL subjects

The third inclusion criterion is that the curriculum of the program should include courses from at least two TEL-relevant domains. For example, a relevant program could include subjects related to education and technology or a relevant program could include subjects related to technology and TEL subjects. At the same time, we considered a program irrelevant if it included subjects related to only one of the TEL-relevant domains listed above.

Finally, the fourth inclusion criterion is that more than 50% of the courses of the program curriculum should belong to one of the TEL-relevant domains. We introduced this criterion to filter out programs that are dominated by subjects from domains other than those defined TEL-relevant.

In order to complement the analysis, we later also included programs provided in languages spoken by the authors of this study, i.e., Norwegian, German and Dutch. This was done to arrive at a broader and richer corpus of programs. We considered that the value of this addition would be greater than the detriment of the introduced noise.

#### Materials and procedures

In our data collection, we followed guidelines for systematic review and meta-analysis (Moher et al., 2009). Our systematic analysis started with an identification of suitable online portals for data collection. We searched for master’s programs in four online portals that aggregate study programs from around the world: Findmasters (https://www.findamasters.com), Masterportal (https://www.mastersportal.com), Masterstudies (https://www.masterstudies.com), and Master and more (https://www.master-and-more.eu/). The data collection was done between May and November 2019.

**Fig. 1:**
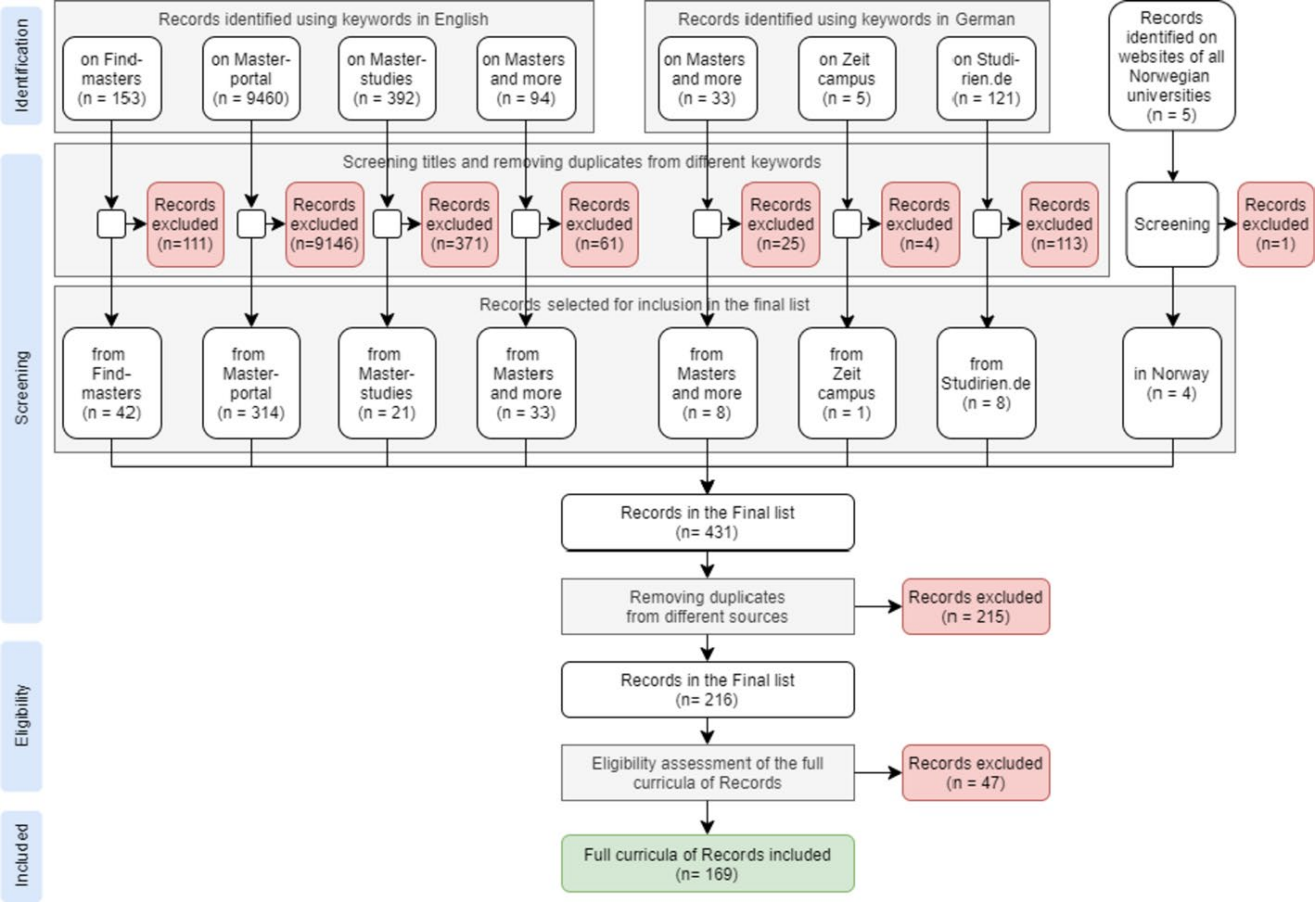
Systematic search process according to PRISMA statement (Moher et al., 2009)

In the *identification phase*, we started the analysis by collecting master’s programs that fulfilled our selection criteria mentioned above (Fig. 1). This initial review of the search results was done by the main author together with two research assistants. At first, the search process was conducted together to resolve possible disagreements, but at a later stage it was carried out independently. It was decided to include the results for discussion in cases of unclarity. We used the same list of keywords to search for master’s programs on all four online platforms: “Technology-Enhanced Learning”, “Educational Technology”, “Computer-based learning”, “Learning with ICT”, “eLearning”, “Learning Innovation”, and “Learning Leadership Innovation''. It should be noted that the numbers of search parameters on different portals were different. We applied additional filters to narrow down the search results so that they better fit the selection criteria whenever possible. For example, on Masterportal, we filtered out programs in the irrelevant subject areas, because the number of search results was too high. For each program, we collected the title, abstract or short description, a university and a country where the program was offered.

In the *screening phase*, the titles (and, where needed, also short descriptions) of the programs were reviewed (Fig. 1). We only removed programs that were clearly irrelevant after reading their title and short description. In order to avoid collecting duplicates returned by the same online portal when using different keywords, we created a user profile on each platform and added the relevant results into “Favorites” or “Wish list”. After the initial review of titles and abstracts, the results from each portal were added to a joint list. After that, the duplicates of the same programs found on different online portals were removed. At the next phase, we collected curricula of each program. In most cases, we could find a list of courses on the website of the corresponding universities. The programs for which we could not find the curriculum on the website of the corresponding university were excluded.

In the *eligibility assessment* phase, we reviewed the curricula of the programs in the joint list (Fig. 1). The programs that did not satisfy the TEL-specific third and fourth inclusion criteria were excluded. At this stage, we also reviewed programs with multiple specializations. In most cases, we kept only the most relevant specialization (e.g., a TEL specialization of an Educational Science program). We kept multiple specializations if they focused on different aspects of TEL. Figure 1 depicts the whole search, screening and eligibility assessment process.

Finally, 169 programs were included for coding and categorization of the curricula and topics contained.

#### Development of categories and codes

The initial coding scheme was applied to 20 programs. Codes were taken from existing curricula and from the literature. Coding was performed inductively, starting with the first program in the final list, and adding new codes (or extending or generalizing existing ones) as we went along. This process yielded a total of 36 codes. Together with the two research assistants, these codes and reasons for eligibility were reviewed to ensure a common understanding so that the remaining programs were coded by the assistants. Some of the programs were not fully coded by the assistants because they were uncertain about some of the technology-related aspects of curricula. This has been resolved by the first author. At this stage, after coding all programs, we arrived at 42 codes.

In the next round, the first author again went through all programs, and minor errors and inconsistencies were corrected. Three composite codes were split, so that from each of them, two new codes appeared. For example, "mobile learning and social media" was split into "mobile learning" and "social media". The code “thesis” was present in 63 programs, but we decided to remove it, as delivering a thesis is required for a master’s degree, and it does not carry content-related information. Some universities explicitly mention thesis or dissertation as part of their curricula, while others do not. After this process, we arrived at the final 44 codes.

In addition, we marked each code as either “core” (for courses labeled core, emphasis, TEL specializations or unspecified) or “elective” (courses labeled elective, support, additional topics or non-TEL specializations). If this information was not available, we considered the courses to be of the “core” category. Where a program contained multiple courses matching the same code and at least one of the courses was of the “core” category, we marked the code as “core”.

### Phase 2: Hierarchical cluster analysis

#### Data preparation

The final list of programs produced in the first phase was taken as input for the second phase of the analysis. For this purpose, the initial list was cleaned from extraneous information so that only topics and programs were contained in the data matrix. Then, the matrix was converted to a binary matrix so that each cell was either filled with a “0” or a “1”.

#### Materials and procedures

A hierarchical cluster analysis of the study programs was conducted. For this purpose, we used R-Studio (Version 1.2.5033) with R (Version 3.5.1). The spreadsheet of all collected programs was imported for analysis. Two different approaches for cluster analysis were applied. Due to binary data, we used the Jaccard distance to compute the similarity matrix for further analysis (Jaccard, 1908). We compared a bottom-up approach (Agglomerative Hierarchical Clustering) and a top-down approach (Divisive Hierarchical Clustering) and evaluated four different methods for comparing the (dis-)similarity of the clusters in the data, of which Ward’s minimum variance method (Ward, 1963) yielded the highest agglomerative coefficient (AC) compared to the average-linkage, the single-linkage and the complete-linkage approach.

To determine the best number of clusters for further analysis, we employed a gap-analysis (Tibshirani et al., 2001) and produced a silhouette plot (Rousseeuw, 1987). A two-cluster solution was followed by a three-cluster solution for the silhouette width analysis and the gap analysis. After controlling for face-validity and interpretability, we also tested a three-cluster solution and used this for further analysis. After inspecting the resulting dendrograms of both clustering approaches, we decided to retain the agglomerative hierarchical clustering approach.

After transposing the data matrix, a second hierarchical cluster analysis of the topics contained in curricula of the analyzed programs was conducted using the same toolset and approach. Due to multiple options for clustering, we tested a 2-cluster, 4-cluster and 8-cluster solution and discussed face-validity of the different visualization. We finally selected the 4-cluster solution as the one which had the best interpretability and followed the same agglomerative hierarchical clustering approach as we have used in the first clustering analysis.

## Results

The results will be presented according to the main phases of the study. First, we present the general results of the systematic search. Second, we present the clustering results, including an analysis of programs and an analysis of topics. Finally, we present a bulk of results addressing the core versus elective topics.

### Systematic search results

The numbers of results for different English keywords returned on different platforms are presented below (Table 1). The left part of Table 1 shows the number of search results returned for each keyword independently before screening. Across the four portals, we received 10,099 results. The right part of Table 1 shows the number of programs retained after the initial screening phase, described in Fig. 1 above (review of the titles and where needed also the short descriptions).

**Table 1:** Number of search results for english keywords

Keyword	Number of search results							
	After the identification phase				After the screening phase			
	Find-masters	Master-portal	Master-studies	Master and more	Find-masters	Master-portal	Master-studies	Master and more
“Technology-Enhanced Learning”	23	683	20	5	10	73	3	0
“Educational Technology”	25	4310	44	56	19	167	13	17
“Computer-based learning”	4	536	120	0	0	11	2	0
“Learning with ICT”	0	176	11	8	0	9	0	3
“eLearning”	100	34	113	25	12	11	2	13
“Learning Innovation”	1	2941	30	–	1	29	0	–
“Learning Leadership Innovation”	0	780	54	–	0	14	1	–
Total per portal:	153	9460	392	94	42	314	21	33
Total per phase:	10,099				410			

The keyphrase “Educational Technology” provided the highest number of results both after the identification phase and after the screening phase (216 programs). However, the share of programs we kept after the screening phase was average. We kept 4.87% of the 4435 programs obtained with “Educational Technology” keyphrase.

Keyphrases “Learning Innovation” and “Learning Leadership Innovation” provided the second and third highest number of results in the identification phase. However, for these two keyphrases, the share of programs that we kept after the screening phases was the lowest. We kept only 1.01% of the 2972 programs obtained with “Learning innovation” and 1.80% of the 834 programs obtained with “Learning Leadership Innovation”.

Keyphrases “Technology-Enhanced Learning” and “eLearning” provided average results after the identification phase. However, for these two keyphrases, the share of programs that we kept after the screening phase was the highest. We kept 11.76% of the 731 programs obtained with “Technology-Enhanced Learning” and 13.97% of the 272 programs obtained with “eLearning”.

In parallel, we received 159 results using keywords in German from three online portals. We also found five results by browsing all master programs in all universities in Norway. These results are not shown in Table 1.

#### General results

The most popular titles of the programs are Educational Technology (44 programs), Instructional Design and Technology (16), Instructional Technology (11), and Education Technology (6). The titles of the other 92 programs appear once or twice. The titles of the programs consist of various combinations of 47 keywords, from which the most popular are: Technology (58 appearances of the keyword in the titles), Education (39), Learning (30), Instruction (16), Media (12), Design (11), Digital (10), Communication (8), Leadership (5), Teaching (5), E-Learning (4), Management (4), and Training (3). Other keywords appear in less than three programs.

Most of the selected programs (65%) are offered in the US, followed by 15% of programs in continental Europe, and 10% in the UK. The rest of the selected programs (10%) are offered in Asia, the Americas excluding US, and Australia.

**Fig. 2:**
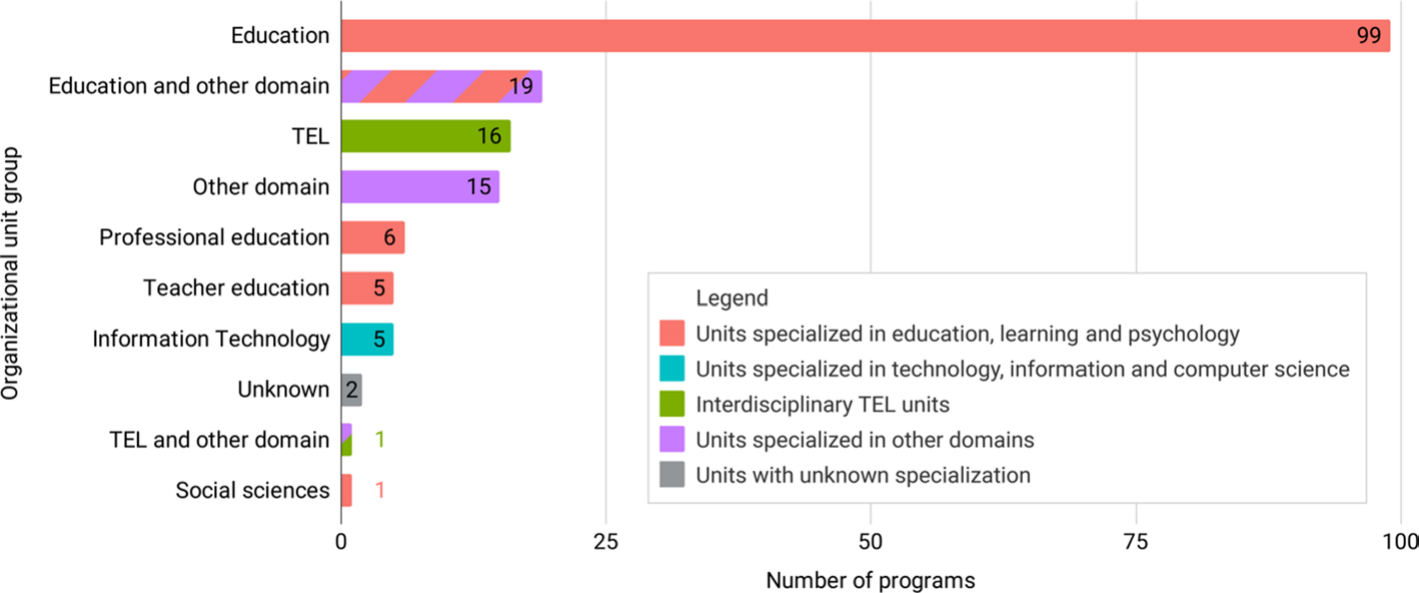
Organizational units that offer TEL programs

We collected the names of the organizational structure units (such as faculties, departments, schools, colleges) where the selected programs are offered. We started by collecting the names of the highest organizational units (such as faculty or college). Where the information was available, we replaced the names with the lower organizational structure units (such as department or institute). We categorized the units into groups (Fig. 2). The largest group contains 99 programs (59%) that are offered by units, focusing on education. The typical units in this group would be entitled “Department of Education” and “College of Education”. The second largest group contains 19 programs (11%) offered by units, focusing on education together with other disciplines, but not TEL. Examples of these units are “College of Education and Health Sciences” or “College of Education & Integrative Studies”. The third largest group of 16 programs (9%) are offered by interdisciplinary units in TEL, such as “College of Education, Information & Technology” or “Department of Computer Education and Instructional Technology”. The fourth largest group of 15 programs (9%) are offered by units that specialized in domains other than education, IT or TEL. The following two groups are Professional education and Teacher education. Disciplinary units focused on technology and computer science offer five of the selected programs (3%).

We collected the master’s degree types granted at the successful completion of the selected programs. The most common type of master’s degree granted in the selected programs is Master of Arts (33%), closely followed by Master of Education (30%), and Master of Science (27%). A degree Master of Arts in Education (that we sometimes find in the US) is granted in 2% of the programs. The rest of the programs (8%) award either uncommon or unknown forms of master’s degree.

**Fig. 3:**
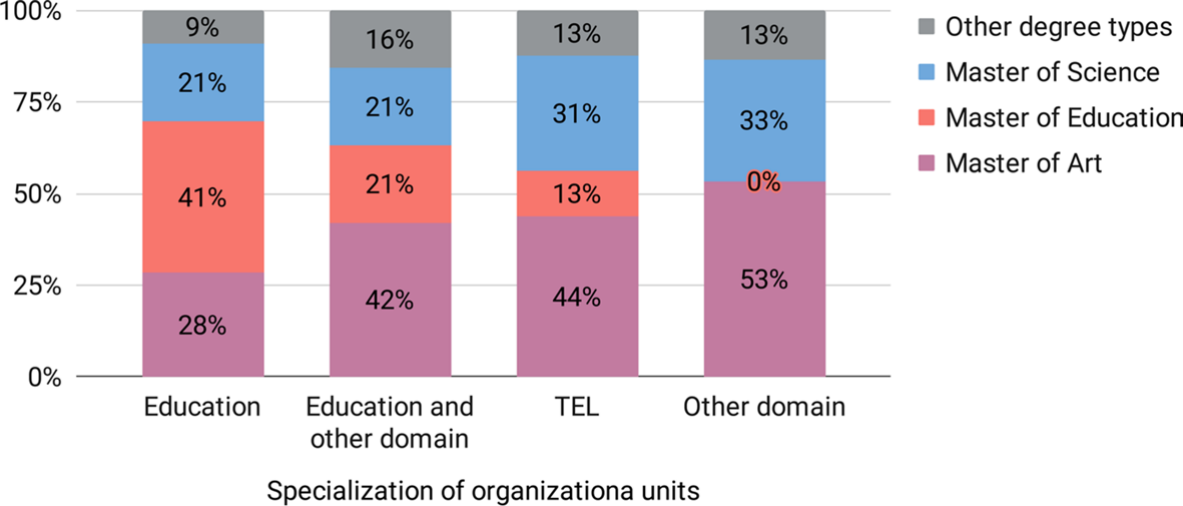
Degree types of the four largest groups of organizational units

Notably, the organizational units of education (Fig. 3) most often offer the degree of Master of Education (41%), followed by Master of Art (28%). The organizational units of education together with other disciplines offer most often the degree Master Art (42%) and equally common (21%) the degrees Master of Education and Master of Science. At the same time, the organizational units in TEL most often offer the degree Master of Art (44%), followed by Master of Science (31%).

We collected the data on the delivery mode or modes of the selected programs. For each program, we looked at if it is offered on campus, online or in a blended mode. We also tried to capture if the programs are offered in different modes, for example, if the same program is offered either on campus or online (although, in some cases, program descriptions do not mention this explicitly). We did not collect the data to distinguish the day- and evening-delivery modes, as the only a small part of the program descriptions explicitly describe this.

A large number of the selected programs (41%) is offered in an on-campus delivery mode. The next largest group (29%) consists of programs offered fully online. The third largest group (14%) consists of programs offered in a blended mode. Among the programs that are offered in different modes, either on campus or online is the most common combination (9%). The combination of alternatives blended or online is offered by 2% of the programs. All three alternatives (on campus or blended or online) are offered by 1% of programs. We could not identify the delivery modes of the remaining 4% of the programs.

#### General curricula results

**Fig. 4:**
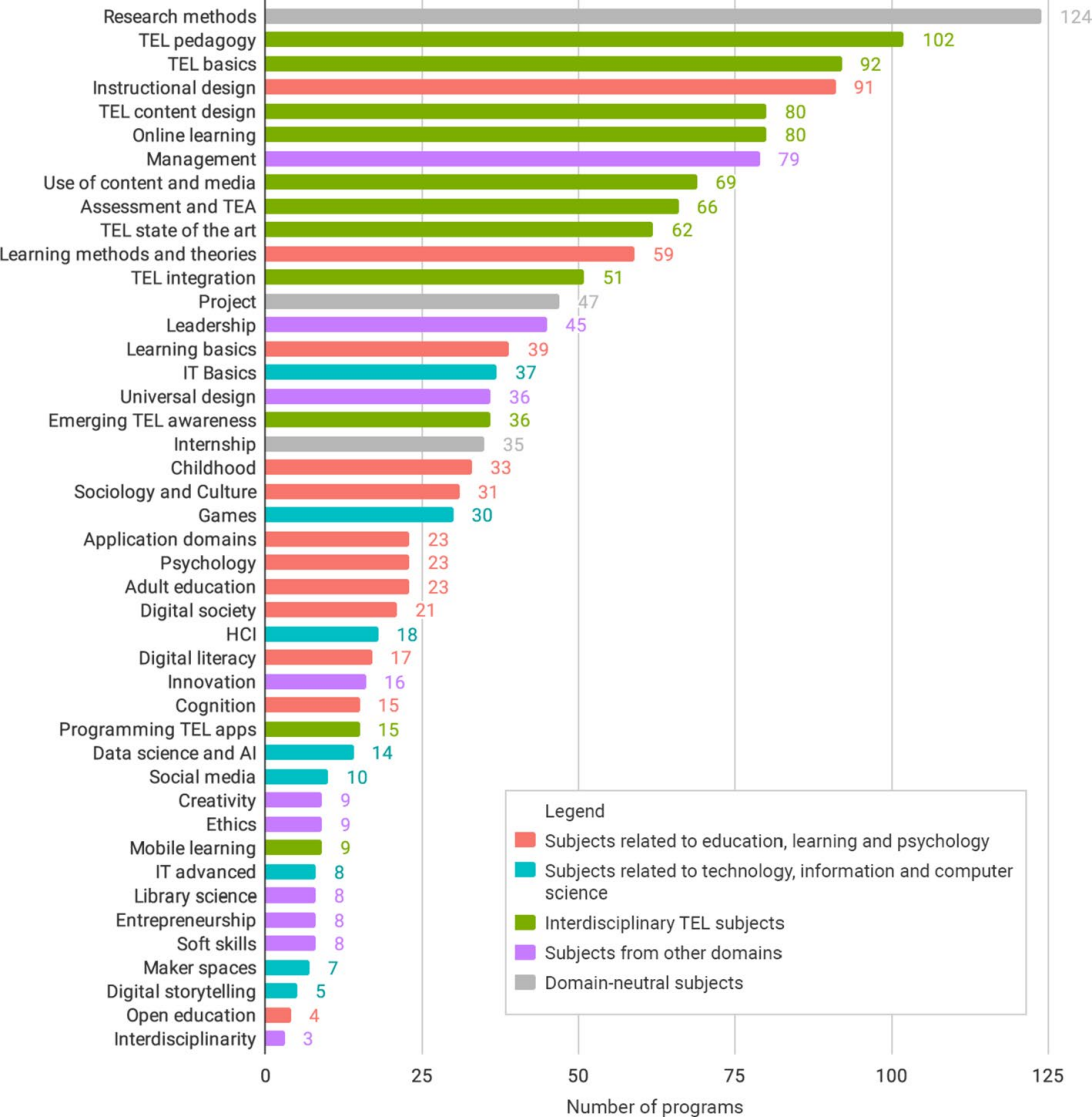
Frequency of topics in TEL curricula

The curricula of the identified programs were coded with the following 44 topics. Most of these topics were associated with the three TEL-relevant domains that we defined in the search phase. For the topics from other disciplines that could not be included in these domains, we defined a new domain “Other”. In addition, the programs included three domain-neutral topics (Fig. 4). The descriptions of each topic can be found in the Table in Annex.

Figure 4 above shows the frequency of the appearance of the topics in TEL master programs. The topics of domain 3 “Interdisciplinary TEL subjects” appear in TEL master programs most often (662 times across the 11 topics). Domain 1 “Subjects related to education, learning and psychology” is represented by 12 topics that appear 379 times in the programs we selected in this study. The 10 topics from the new ‘Other’ domain appear 221 times. The three domain-neutral topics appear 206 times. Finally, the eight topics of Domain 2 “Subjects related to technology, information and computer science” appear 129 times.

### Clustering results

During the initial analysis of the program cluster solutions, we identified several outliers, which could potentially bias the cluster analysis. For this reason, we calculated the mean number of topics in the sample (9) and deleted all programs with less than six topics. After deleting 15 programs, we continued the cluster analysis with 154 programs.

#### Clustering of TEL programs

**Fig. 5:**
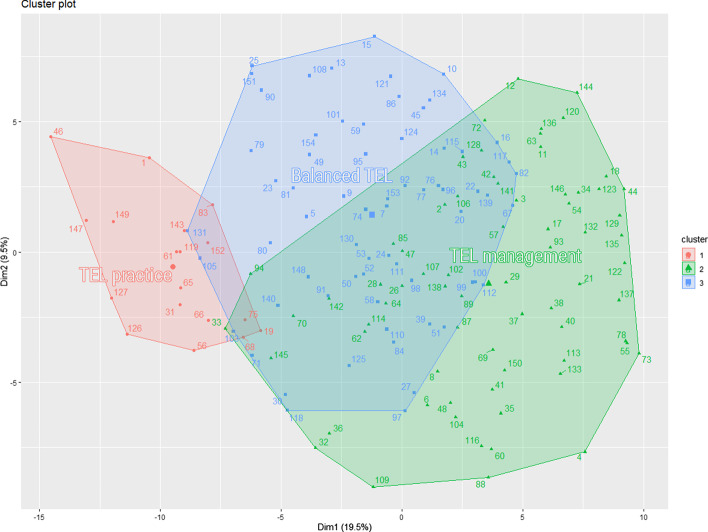
Visualization of hierarchical cluster analysis of 154 study programs on TEL

Results of the clustering of the remaining TEL programs are presented in Fig. 5. As we can see from this figure, there are three large clusters of programs, and we can identify six different positions of programs in this visualization. Programs either belong to the red cluster (18 programs), to the blue cluster (66 programs) or to the green cluster (70 programs). Alternatively, they fall into the intersection of two clusters or even in the intersection of all three clusters.

Cluster 1 in red contains 18 programs that focus on the practical subjects of designing and using TEL systems and content. This cluster can be called TEL practice. The programs in this cluster rely on subjects from all three domains pre-defined in the study. A set of TEL topics common in this cluster include, ordered here by the frequency of their appearance: *TEL basics*, *Use of content and media*, *Emerging TEL awareness*, *TEL integration*, *TEL pedagogy*, and *Online learning*. The common topics of this cluster include two introductory topics from the educational science (*Learning methods and theories* and *Learning basics*) and two topics from the technology domain (*Games* and *IT Basics*).

**Fig. 6:**
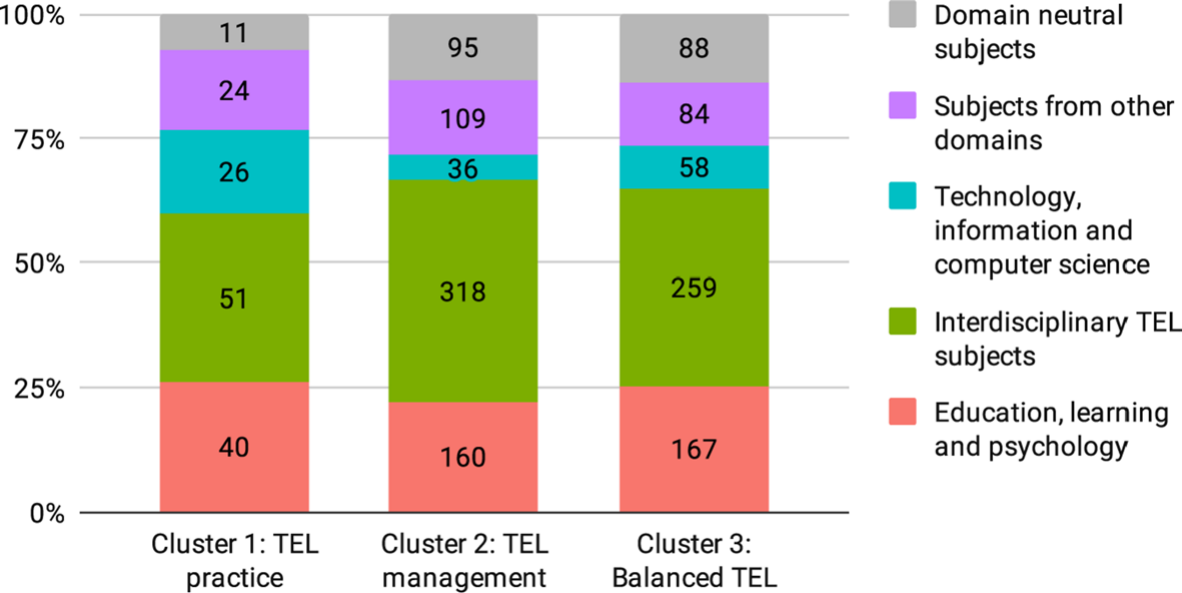
Share of topic domains in the Program clusters

This cluster is also characterized by a larger share of topics from the technology domain than the other two clusters (Fig. 6). Programs in the TEL practice cluster stand out from the other two clusters by a higher number of the following topics: *Data science and AI*, *Entrepreneurship*, *Social media*, *IT advanced*, *Open education*, *Innovation*, and *Games*.

Cluster 2 in green contains 70 programs that focus on the organizational management of the TEL processes. This cluster can be called TEL management. Programs in this cluster distinctly rely on subjects that were coded with the topics of *Management* and *Leadership*. From the educational domain, the programs in this cluster include *Instructional design* much more often than any other topic and also more often than in the other clusters. Programs in the TEL management cluster also rely on a set of TEL topics, more than the other two clusters (Fig. 6). The TEL topics most common in the TEL management cluster are, ordered here by the frequency of their appearance: *Online learning*, *TEL pedagogy*, *TEL state of the art*, *TEL basics*, *Assessment and TEA*, *TEL integration*, *TEL content design*, and *Use of content and media*. This cluster also contains programs with the largest share of the domain neutral topic *Research methods*.

Programs in the TEL management cluster stand out from the other two clusters by a higher number of the following topics: *TEL state of the art*, *Leadership*, *TEL integration*, *Management*, *Online learning*, *Instructional design*, *Assessment and TEA*, *Adult education*, and *Research methods*. The cluster also exclusively contains all subjects coded with *Interdisciplinarity* and *Library science*.

Cluster 3 in blue contains 66 programs that focus on TEL subjects and include varied technological and educational subjects. This cluster can be called Balanced TEL (Fig. 6). The programs in this cluster contain a set of TEL topics, ordered by the frequency of their appearance: *TEL pedagogy*, *TEL content design*, *Use of content and media*, *TEL basics*, *Assessment and TEA*, *Online learning*, and *Emerging TEL awareness*. The programs in the cluster also include foundational subjects from both the technological and the educational domain (*IT Basics*, *Instructional design*, *Learning methods and theories*, and *Learning basics*). The programs in this cluster include a considerable share of technological subjects (*Maker space*, *HCI*, and *IT Advanced*) and one more technology-focused TEL topic *Programming TEL apps*. This cluster also contains programs with the largest share of the domain neutral topics *Project* and *Internship*.

Programs in the Balanced TEL cluster stand out from the other two clusters by a higher number of the following topics: *Programming TEL apps*, *Universal design*, *Maker spaces*, *HCI*, *Cognition*, *Application domains*, *IT Basics*, *IT advanced*, and *Emerging TEL awareness*. This cluster also almost exclusively contains subjects coded with *Ethics* and *Soft skills*.

The intersections of clusters provide additional insights. The largest intersection area contains 24 programs from Cluster 2 TEL management and 40 programs from Cluster 3 Balanced TEL (Fig. 5). The programs in this intersection area include mostly foundational TEL subjects and foundational education subjects. The programs in this area do not have as much focus on the management and leadership subjects as in the TEL management cluster. At the same time, the programs in this area do not have as much focus on the technological subjects as in the Balanced TEL cluster.

Other smaller intersection areas do not highlight major differences or do not contain enough programs to make conclusions.

#### Clustering of TEL topics

The second hierarchical cluster analysis of curricula resulted in four topical clusters contained in these programs.

**Fig. 7:**
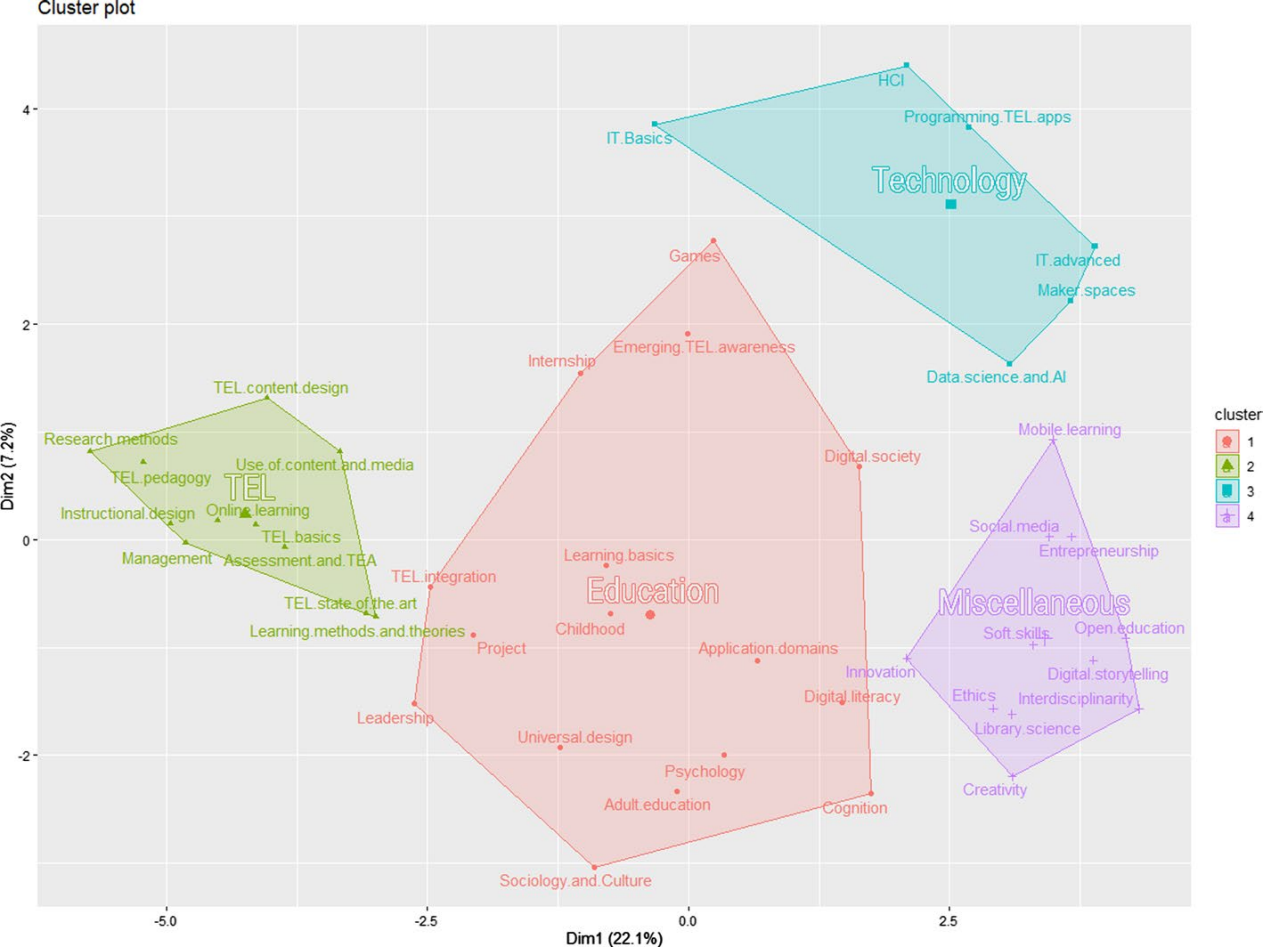
Visualization of hierarchical cluster analysis of 44 curriculum topics identified in study programs on TEL

**Fig. 8:**
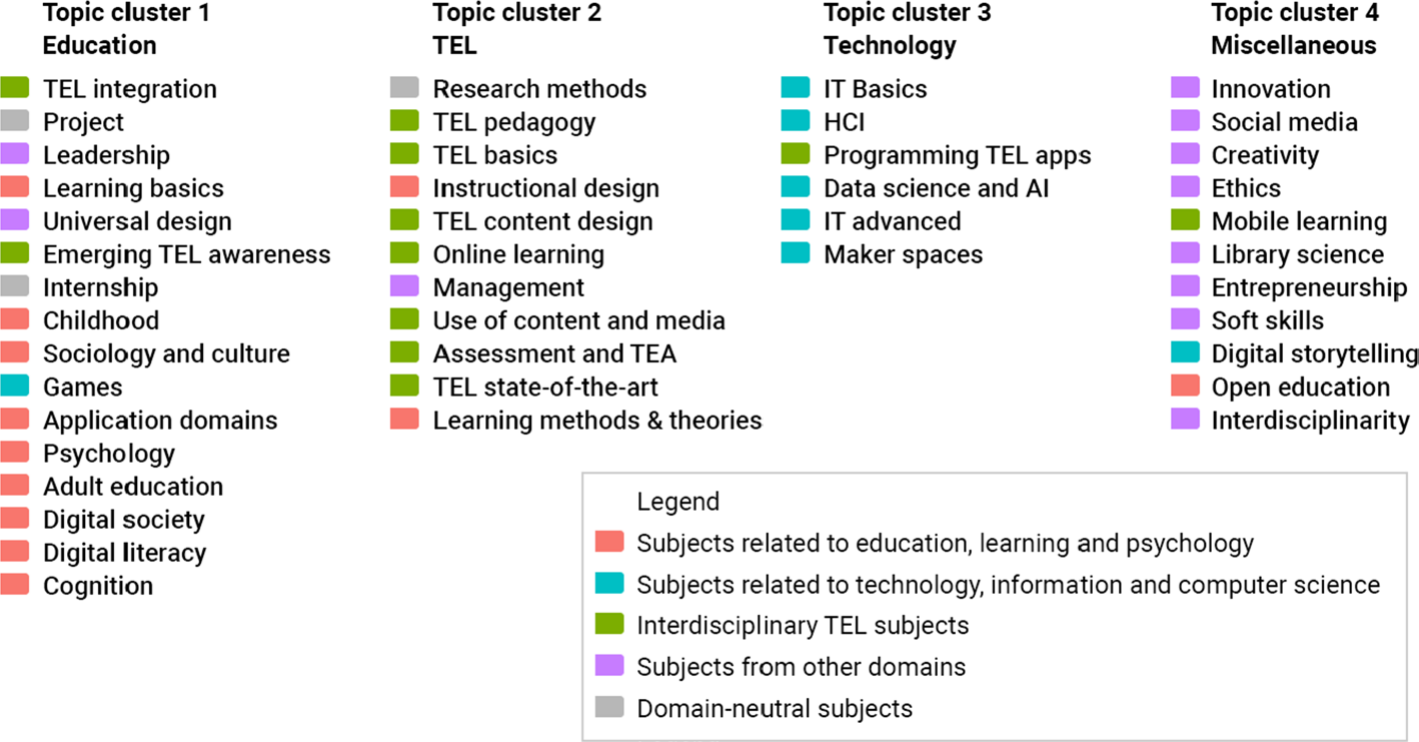
Domain affiliation of topics in Topic clusters

The clustering solution led to four independent topic clusters (Fig. 7). The topic clusters are named based on the majority of subject each of them contains (Fig. 8).

Topic cluster 1 “Education” in red contains 16 topics. Nine of these topics were earlier classified to Domain 1 “Subjects related to education, learning and psychology”. The cluster also contains one or two topics from other domains.

Topic cluster 2 “TEL” in green contains 11 topics. Seven of these topics are from Domain 3 “Interdisciplinary TEL subjects”. The cluster also contains two key topics from Domain 1, one domain-neutral topic, and one topic from the “Other” domain.

Topic cluster 3 “Technology” in cyan contains six topics. Five of these topics belong to Domain 2 “Subjects related to technology, information and computer science”. The cluster also contains one topic that was originally classified to Domain 3 but required advanced IT knowledge.

Topic cluster 4 “Miscellaneous” in violet contains 11 topics. Seven of these topics belong to the new domain “Other”, which includes topics that appear in TEL programs but do not fit the three pre-defined domains. The Miscellaneous cluster also includes one or two topics from domains 1, 2 and 3.

### Core-elective

In the cluster analyses, we did not distinguish between core (for courses labeled core, emphasis, major, TEL specializations or unspecified) and elective (courses labeled elective, support, minor, additional topics or non-TEL specializations) subjects in the data. These data were analyzed separately, after the cluster analysis. Among the 169 programs, only 52 mentioned which subjects are core and which are elective. We particularly looked at the core-elective data for the 154 programs that were included in the cluster analysis. Just below one third of them (50) mentioned elective subjects of at least one topic.

The topics that were most often included as “core” are: *TEL basics* (core in 96% of programs), *Research methods* (95%), *Project* (94%), *Learning basics* (92%), *Instructional design* (91%), *TEL integration* (90%), and *TEL state of the art* (90%). The topics that were most often included as elective are: *IT advanced* (core in 63% of programs), *Maker spaces* (71%), *Games* (70%), *Data science and AI* (71%), *Creativity* (67%), *Application domains* (57%), *Mobile learning* (44%), and *Open education* (50%). It should be noted that the ratio of appearances as core for a single topic ranged from 44 to 96%, mostly because many programs did not mention elective, and we considered all their subjects as core.

For each topic, our data showed in which programs it appeared as an elective. We first made a breakdown of how often each topic appeared as elective in each of the three clusters of programs. At the next step, we summed the values of the topics in each of the four topic clusters. Finally, we converted the numbers of appearance to percentages. These steps resulted in a table that shows how often topics from each topic cluster appear as core or as elective in each of the program clusters (Table 2).

**Table 2:** The ratio of core and elective topics from each topic cluster in each program cluster

	Share of core topics		
	Program cluster 1 TEL practice (%)	Program cluster 2 TEL management (%)	Program cluster 3 Balanced TEL (%)
Topic cluster 1 Education	94	81	78
Topic cluster 2 TEL	95	86	88
Topic cluster 3 Technology	78	63	83
Topic cluster 4 Miscellaneous	94	77	68

From the table above, it is clear that Program cluster 1 TEL practice includes core topics from Topic clusters 1 Education (94%), 2 TEL (95%), and 4 Miscellaneous (94%), while the topics from Topic cluster 3 Technology are more often elective.

Program cluster 2 TEL management includes core topics from Topic cluster 2 TEL moderately often (86%), while topics from Topic cluster 3 Technology appear as elective very often.

Program cluster 3 Balanced TEL includes core topics from Topic cluster 2 TEL moderately often (88%) and Topic cluster 3 Technology slightly less often (83%). At the same time, this program cluster often includes topics from Topic cluster 4 Miscellaneous as elective.

Moreover, it can be seen that topics from Topic cluster 2 TEL appear as core most often in all three program clusters.

## Discussion

This study was conducted to provide an overview of the state of the art in education in TEL at master level. In particular, we aimed to identify the general characteristics of TEL study programs (RQ1), the composition and differences in TEL curricula (RQ2), and the organization of course topics in TEL master programs (RQ3). The discussion of the results will lead to the practical implications for the development of study programs in TEL, theoretical and methodological contributions of the article.

### Characteristics of Technology-Enhanced Learning master programs

To answer our first research question *RQ1: What are the characteristics of TEL master programs?*, we provide an overall discussion of descriptive results.

With regard to the distribution of institutions which offer these programs the majority is located in the US followed by Europe and the UK. This is not surprising given that we focused on programs offered in English. At the same time, it is informative to identify the ratio and geographical distribution between study programs with regard to future development of the field. The vast majority of programs were located in organizational units related to education and only a small fraction was offered by other units. This is an interesting result especially with the interdisciplinarity of the TEL field in mind (Kalz & Specht, 2014) and also with recent results of bibliographic studies on TEL which show a larger contribution from computer science (Shen & Ho, 2020). This ratio of contributing domains is not represented in the educational offer because the majority of institutionalized formats in TEL still reside in Education as a core domain and not, for example, in computer-science or psychology where education functions as a service domain.

Fittingly, Master of Arts is the most awarded degree in our sample of programs, given it is the broadest in scope and likely encompasses educational programs as well. Similarly popular, the Master of Education degree also fits well with the dominance of education units that grant these degrees. However, in light of this, it is notable that almost one-third of programs grant a Master of Science degree. The type of degree often follows the institutional profile and practice of the organizational unit that offers a study program. From the results of the study, we can see that the organizational units that position themselves within the discipline of education, offer the Master of Education degree most often, and in some cases—Master of Art, which might be the consequence of practice or tradition. The organizational units that position themselves as multidisciplinary (education together with other disciplines, but not technology) offer the Master Art degree much more often, which implies that this degree covers a broad range of disciplines.

On this background, it is most notable to see that the organizational units that position themselves in TEL most often offer the Master of Art and Master of Science degrees. The Master of Education degree is awarded much less commonly by such units. This might indicate that when an organizational unit is defined by the field of TEL, the educational component is less prevalent than overall.

The term “Educational Technology” is dominant, especially in the US, when describing the TEL master programs. This term describes the TEL master programs with good accuracy, but low precision, as it appears in the descriptions of many master programs that do not focus on TEL. Therefore, this term is somewhat overused. Two other terms “Technology-Enhanced Learning” and “eLearning” are used less often than “Educational Technology”. However, these terms describe the TEL master programs with a higher precision, as they appear much less often in descriptions of programs that do not focus on TEL.

Our findings regarding the delivery modes of teaching suggests that on-campus teaching remains the preferred approach, with most programs exclusively providing this traditional delivery mode. However, there is also a substantial share of programs offering fully online and a smaller set providing blended forms of teaching. Given the substantial overlap between the expertise of TEL researchers and the practical aspects of using technology for teaching purposes, it appears plausible that TEL programs would more readily offer their teachings in “innovative” delivery modes of fully online and blended learning, especially after the Covid-19 pandemic. However, there is currently no research that can attest to this by comparing research fields in this way.

To further discuss the characteristics of master programs in TEL, we look at the general curricula results in the beginning of the next section.

### Curricula of Technology-Enhanced Learning master programs

The general curricula results (section “General curricula results” and Fig. 4) demonstrate that master programs in TEL rely primarily on interdisciplinary TEL subjects (662 subjects, 11 topics) and secondarily on subjects related to education, learning and psychology (379 subjects, 12 topics). Further, master programs in TEL contain relatively few subjects related to technology, information and computer science (129 subjects, 8 topics). We can also see that master programs in TEL contain a considerable number of subjects classified as ‘Other’ (221 subjects, 10 topics) which do not belong to any of the three relevant domains defined in the beginning of the study. Therefore, this analysis of curricula content further supports our findings regarding the dominance of education over computer science in TEL programs in terms of organizational structure units and degree types.

Furthermore, the general results demonstrate that almost all TEL master programs include interdisciplinary TEL subjects. This implies that the subject-level integration is common. In contrast, only two programs out of 169 rely on a program-level integration, not including any interdisciplinary TEL subjects, but a combination of subjects from education, learning and psychology and subjects from technology, information and computer science.

To answer our second research question *RQ2: How distinct are TEL master programs in terms of their curricula?*, we identified three clusters of programs (section Clustering of TEL programs, Fig. 5). Programs in all three clusters rely on the multidisciplinary TEL subjects, as can be seen on Fig. 6. At the same time, the clusters have distinct features that provide valuable insights.

Programs in the TEL practice cluster contain subjects that focus on practitioner skills, such as *Use of content and media* from the interdisciplinary TEL topics, *Learning methods and theories* form the education domain, and almost all practical subjects from the technological domain. From the ‘Other’ domain, the programs in this cluster include *Entrepreneurship* and *Innovation*. The share of the technology-related subjects is high but not dominant in this cluster. This is further supported by the analysis of core versus elective subjects in this cluster that shows that the technology subjects are often included as elective. Notably, the TEL practice cluster contains only 18 of the 154 programs that were selected for the cluster analysis. It means that such a practical focus and an almost equal share of technology topics (compared to TEL topics and educational topics) is rather rare.

Cluster 2 in green can be called “TEL management”. Programs in the TEL management cluster rely mostly on a combination of interdisciplinary TEL subjects and subjects from the ‘Other’ domain that focus on the management of TEL in an organization. The interdisciplinary TEL subjects that cover online learning, TEL pedagogy and assessment, TEL basics and state of the art, and the integration of TEL in organizations are more common in this cluster compared to subjects that focus on content design and use.

From the ‘Other’ domain, the programs in the TEL management cluster often include subjects that focus on management (which also includes administration, funding and policy) and leadership. From the educational domain, the programs in this cluster most often include the subject of instructional design. A possible explanation for this is that instructional design focuses on analysis, planning, and design of instruction, in contrast to the hands-on delivery of instruction. The programs in this cluster contain very few subjects from the technology domain. The core versus elective analysis shows that these subjects are mostly included as electives.

From the domain-neutral topics, the TEL management cluster most often includes *Research methods*. This highlights a strong academic focus of the programs in this cluster. Further, it contains 70 programs, which makes it the largest of the three, implying that management of TEL in organizations is a common and popular course of study. It should be noted that overall TEL programs that focus on management tend to include only a single subject from educational sciences *Instructional design* and almost no technology subjects. Instead, such programs often include subjects related to management, administration and leadership.

Programs in the Balanced TEL cluster contain both practical and theoretical subjects from all three relevant domains defined in this study. For example, theoretical TEL subjects *TEL pedagogy* and *TEL basics* are included as often as practical *TEL content design* and *Use of content and media*. The most practical and technology-focused TEL topic *Programming TEL apps* appears mostly in this cluster. Theoretical educational subjects *Learning methods and theories* and *Learning basics* are included together with a more practical subject of *Instructional design* and theoretical technology subjects coded with *IT Basics*.

With 8.8%, the share of technological topics is small, but considerable. Notably, the programs in this cluster include subjects on varied technology topics, which in addition to *IT basics*, include *Maker space*, *HCI* and *IT advanced*. This is the only cluster where the technological subjects are included as core more often than educational subjects. The programs in this cluster contain a considerable number of topics from the ‘Other’ domain, but the share of these topics is smaller than in the other two clusters.

From the domain-neutral topics, the Balanced TEL cluster often includes *Research methods*. However, in contrast to the TEL management cluster, the largest share of the topics *Project* and *Internship* appear in this cluster. This indicates a more practical focus of the programs in this cluster.

Programs in the Balanced TEL cluster combine TEL subjects with both technological and educational subjects. At the same time, the programs do not rely as much on the topics from the other domains. In addition, it can be noted that in the programs of this cluster foundational and overview subjects appear more often than specialized ones.

### Course topics in Technology-Enhanced Learning master programs

To answer our third research question *RQ3: How are course topics organized in TEL master programs?*, we identified four clusters of topics (section Clustering of TEL topics”, Fig. 7). Based on the analysis of the cluster plot on Fig. 7, we propose the following interpretation.

Topic cluster 1 Education in red is located in the center of the plot. The cluster occupies the largest space, and the topics appear far from each other. It contains most subjects related to education, learning and psychology. A single technology topic of the cluster *Games* appears in the top of the cluster, close to Topic cluster 3 Technology. Further, the cluster includes two TEL topics. More technology-focused *Emerging TEL awareness* also appears in the top part of the cluster, while a more managerial *TEL integration* appears on the left, close to Topic cluster 2 TEL. Two topics from the ‘Other’ domain were included in this cluster. Another managerial topic *Leadership* appears also on the left side of the cluster, while *Universal design* (which also includes subjects related to accessibility, inclusion, and diversity) appears in the center. Two domain-neutral topics *Project* and *Internship* appear in this cluster.

Topic cluster 2 TEL in green is located in the far left of the plot. The cluster is small, and the topics are close together. Every topic of cluster 2 appears in TEL programs more frequently than any topic of other clusters. The cluster contains most interdisciplinary TEL subjects together with two educational topics: *Learning methods and theories* and *Instructional design*, one domain-neutral topic *Research methods*, and *Management*—a single topic from the ‘Other’ domain.

Topic cluster 3 Technology in cyan is located in the top right part of the plot. The cluster is relatively large, and the topics are far from each other. It contains most technology-related topics and a single TEL topic *Programming TEL apps*. The topic of *IT basics* is on the far left of the cluster, closest to Topic cluster 1 Education, which can be interpreted as this topic is often introduced in programs that include mostly educational subjects.

Topic cluster 4 Miscellaneous in violet is located in the bottom right of the plot. The cluster is relatively small, and the topics appear close together. It contains most subjects from the ‘Other’ domain. A single TEL topic in this cluster *Mobile learning* appears on the far top of the cluster, closer to Topic cluster 3 Technology. The cluster also contains two technological topics *Social media* and *Digital storytelling* and a single educational topic *Open education*.

A general trend visible in topic cluster plot (Fig. 7) is that the vertical axis represents the scale from practical topics in the top to theoretical and organizational in the bottom. For example, in the Education cluster *TEL content design* is on the top, while *Learning methods and theories* is in the bottom. The horizontal axis reflects the subject domains.

The analysis of core versus electives, presented in section “Core-elective”, indicates that the Education cluster contains subjects that appear most often as core courses rather than elective. The TEL cluster contains subjects that appear more often as elective, but still mostly as core. The topics of the Technology cluster appear even more often as elective, while the topics of the Miscellaneous cluster appear as elective most often.

The highest share of elective subjects in the Miscellaneous cluster appears logical and confirms the choice of our three TEL-relevant domains. These subjects are included as auxiliary and complementary to the main curricula. Still, we found that subjects on these specific topics are included in the studied TEL master programs.

The relatively high share of elective subjects in the Technology cluster is in line with the general curricula results. In addition to the subjects of this cluster often being included as electives, they are included in TEL programs less often than interdisciplinary TEL subjects and educational subjects.

The subjects of the Education cluster and the TEL cluster appear most often as core and less often as elective. This result is in line with the general curricula results. In addition to the subjects on these two topic clusters often being included as core, they are most often included in TEL master programs.

However, the results that subjects of the Education cluster appear more often as core than those of the TEL cluster is unexpected. All studied TEL programs included subjects from at least two out of three relevant domains (third inclusion criteria, section “Sample definition”). The lowest percentage of elective subjects in the Education cluster indicates that educational subjects almost always play the key role in the TEL curricula, regardless of what other subjects are included.

## Conclusions

The study presented in this article provides a description and analysis of the most important characteristics of master-level education in the interdisciplinary field of TEL. Among other details, it reveals what topics are considered to be important and allows to understand what competences and skills can be expected from the graduates. The in-depth analysis of the curricula highlights the common trends and the differences of the study programs, providing practical implications for designing new and developing existing programs.

We can conclude that the major similarity of the TEL master programs is the dominance of interdisciplinary TEL and educational subjects in their curricula. The major distinguishable features of the master programs are reflected by the three clusters of programs. The largest cluster TEL management includes programs that rely on a combination of subjects that focus on the management of TEL in an organization with a very small share of technological subjects. The second largest cluster Balanced TEL includes programs that focus on both practical and theoretical subjects in TEL, education, and technology. The smallest cluster TEL practice includes programs that focus on practitioner skills in TEL, foundational subjects form education, and almost all identified topics from the technological domain. The analysis identified a clear lack of master programs that focus on technology, where education plays the role of the application domain. Only a few examples of such programs have been identified in the study. Another less evident limitation of the study programs is the relatively small representation of new and emerging TEL trends which are high in the research agenda. Many programs cover the awareness of trends in TEL but the emerging topics themselves (such as games and simulations, open education, innovation in TEL, maker spaces, and artificial intelligence in TEL) rarely appear in the study programs.

We can further conclude that certain topics often appear together in TEL master programs. The analysis of these topics revealed four clusters. These clusters of topics mostly resemble the three TEL-relevant domains that we defined prior to the review of the programs: education, technology, and TEL. At the same time, the topics that did not fit any of the three domains also mostly clustered together. However, the clustering revealed that certain subjects from different domains tend to appear together. Most notably, instructional design appears very often together with TEL integration and management. Other examples are the learning methods and theories that are often taught together with TEL subjects, while the awareness about the emerging TEL is taught together with educational topics. The clustering also identified that the interdisciplinary TEL topics appear together more often than the topics of other domains. This implies that master programs often include sets of TEL subjects, which highlights the complexity of the field.

The study of the landscape of TEL master programs presented in this article provides theoretical implications for the field. Given the assumption that research fields translate what they consider to be important and worthwhile into educational programs, the systematic review and analysis of the TEL programs can teach us about features of the field of TEL itself.

Our results demonstrate that TEL, as an interdisciplinary and heterogenous field of research, is represented by masters programs that, too, encompass a wide range of curriculum contents. In sum, we identified 44 distinct topics covered by the subjects included in the master programs. In addition to the topics related to the disciplines of education and technology that define the field of TEL, we discovered a large number of interdisciplinary TEL topics and other diverse topics, such as *Management*, *Leadership*, *Creativity*, *Innovation*, and *Library science*. This speaks to the breadth of the field. Yet, synthesizing results of the topic clustering, the analysis of core versus elective topics, as well as the institutional affiliations and degree types awarded, our results suggest that the fields of education and learning sciences remain the foundation for many of these programs and, thus, the research field itself. Aside from this broad topical weighting, the high frequency of TEL topics like *TEL pedagogy*, *Online learning* and *TEL content design* suggest that TEL is a unitary research field that consists of more than the mere combination of education and computer science. The most common curriculum topic being research methods speaks to the highly empirical orientation of TEL.

The study presented in this article developed a new methodology for the analysis of study programs, focusing on the interdisciplinarity of the field. The methodology included two major phases. The first phase consisted of a systematic search and review of study programs, where the main inclusion criterion is defined by the domains that overlap to constitute the studied interdisciplinary field. The second phase consisted of two cluster analyses. The clustering of study programs allowed us to identify similarities and differences between programs based on their curricula, while the clustering of course topics allowed us to better understand the structures of study programs and typical and rare combinations of topics. Such methodology can be adapted or further developed in studies of other disciplines.

The major limitations of the study presented in this article include the following. The search was restricted to study programs offered in English. The authors later repeated the search in German and Norwegian. Still, inclusion of study programs offered in other languages is a possible direction for future research.

Another limitation is that the study provides a snapshot of the state-of-the-art but not the dynamics. We do not know if and how the landscape has been changing and therefore cannot predict the development of TEL study programs over time. The data in this study were collected prior to the Covid19 pandemic, which greatly affected educational processes to use more TEL approaches. Therefore, it would be particularly interesting to study the field again after the pandemic is over. There might be a share of study programs that will keep the online or blended delivery modes even after the campuses reopen. More importantly, there might be more emphasis on the topic of online learning.

Finally, the study included only master programs. This limitation was intentionally set to limit the scope of the study.

The major contribution of this article is a presentation of the state-of-the-art in master-level education in the interdisciplinary field of TEL. It also contributes to the debate on the growing number of interdisciplinary study programs in learning and technology that define the field itself. The article also suggests a methodology for analyzing interdisciplinary study programs that can be adopted beyond TEL.

## Data Availability

The datasets generated and/or analyzed during the current study are available in the OSF repository, https://osf.io/muxjt/.

## Annex

See Table 3.

**Table 3:** Descriptions of the TEL curricula topic codes

Topic code	Description	Domain	frequency
Research methods	Research methods	Neutral	124
TEL pedagogy	TEL Instructional design, TEL pedagogy, and pedagogical design of TEL environments	TEL	102
Instructional design	Instructional design, including analysis, planning, and design of instruction	Ed	92
TEL basics	Basics of TEL and TEL conceptualization	TEL	91
Management	Administration of educational process, management, funding and policy	Other	80
Online learning	E-Learning, online learning, and distant learning	TEL	80
TEL content design	Design of educational content and media using authoring tools	TEL	79
Use of content and media	Search, selection and use of educational tools and media content	TEL	69
Assessment and TEA	Evaluation and assessment and technology-enhanced assessment, human performance technology	TEL	66
TEL state of the art	TEL state-of-the-art, introduction to the current issues and trends in the field	TEL	62
Learning methods and theories	Learning methods and theories, usually an overview of behaviorism, cognitivism, and constructivism and/or focus on specific methods and theories	Ed	59
TEL integration	Integration of TEL into the educational process, change management, and outcome assessment	TEL	51
Project	A large semester project, done individually or in a group	Neutral	47
Leadership	Leadership and educational leadership	Other	45
Learning basics	Introduction to learning sciences	Ed	39
IT Basics	Basics of information technology, information and communication technologies, and/or computer science	IT	37
Universal design	A broad range of subjects including socially-oriented inclusion, disability and diversity and also technology-related universal design and accessibility	Other	36
Internship	Internship and placements	Neutral	36
Emerging TEL awareness	Awareness of emerging TEL technologies, approaches and trends, often including the use of new tools, devices and methods	TEL	35
Childhood	Childhood studies, early age and school education	Ed	33
Sociology and Culture	Sociology and culture, including multicultural education	Ed	31
Games	Design and use of computer games and simulations, including game-based learning	IT	30
Psychology	Psychology, psychology of learning, motivation, and emotion in education	Ed	23
Adult education	Adult education, professional development and training	Ed	23
Application domains	Education in specific application domains (such as health education, math education, STEM education, etc.)	Ed	23
Digital society	Sociology and TEL, socio-technical systems, and digital society	Ed	21
HCI	Human–Computer Interaction, interaction design, and user-centered design	IT	18
Digital literacy	Digital literacy, usually including the most basic computer competences and skills	Ed	17
Innovation	Innovation, innovation in TEL, and innovation in education	Other	16
Cognition	Cognition, cognitivism, brain and mind	Ed	15
Programming TEL apps	Technical design and development of TEL applications, programming applications	TEL	15
Data science and AI	Artificial Intelligence, statistics, and data analytics in TEL	IT	14
Social media	Social media tools and use of social media in education	IT	10
Creativity	Creativity and creativity in TEL	Other	9
Mobile learning	Mobile learning, an learning approach assuming leaning anywhere and using mobile devices	TEL	9
Ethics	Ethics, educational ethics, research ethics and academic integrity	Other	9
Library science	Library and media, classification, cataloging	Other	8
Entrepreneurship	Entrepreneurship	Other	8
Soft skills	Soft skills (also known as 21-century skills)	Other	8
IT advanced	Advanced topics of information technology, information and communication technologies, and/or computer science, mostly related to the design of applications	IT	8
Maker spaces	Maker spaces and design thinking	IT	7
Digital storytelling	Digital Storytelling and presentation	IT	5
Open education	Openness in TEL, open education	Ed	4
Interdisciplinarity	Concept of interdisciplinarity and interdisciplinary teaching	Other	3

## Abbreviations

TEL: Technology-enhanced learning
RQ: Research question
AC: Agglomerative coefficient
IT: Information technology
TEA: Technology-enhanced assessment
AI: Artificial intelligence
HCI: Human–computer interaction

## References

[CR1] Arrington TL, Darabi A (2018). Indicators of exemplary programs in instructional design and technology: Faculty and student perspectives. Educational Technology Research and Development.

[CR2] Bayne S (2015). What's the matter with ‘technology-enhanced learning’?. Learning, Media and Technology.

[CR3] Bourdeau, J., & Balacheff, N. (2014). Technology-Enhanced Learning: From thesaurus and dictionary to ontology. Dans Jovanovic, Jelena et Chiong, Raymond (dir.), *Technological and Social Environments for Interactive Learning* (p. 1–33). Informing Science Press.

[CR4] Conole, G., Scanlon, E., Mundin, P., & Farrow, R. (2010). Technology-enhanced learning as a site for interdisciplinary research. Technology Enhanced Learning Research Programme; Economic and Social Research Council; Institute of Education, London.

[CR5] Dennen VP, Spector JM (2007). Preparing educational technology leaders: Reflections on the past, present, and future. Educational Technology.

[CR6] Hartley R, Kinshuk K, R., Okamoto, T., & Spector, J. M. (2010). The education and training of learning technologists: A competences approach. Journal of Educational Technology & Society.

[CR7] Ivanitskaya L, Clark D, Montgomery G, Primeau R (2002). Interdisciplinary learning: Process and outcomes. Innovative Higher Education.

[CR8] Jaccard P (1908). Nouvelles recherches sur la distribution florale. Bulletin De La Societe Vaudoise Des Sciences Naturelles.

[CR9] Kalz M, Specht M (2014). Assessing the crossdisciplinarity of technology-enhanced learning with science overlay maps and diversity measures. British Journal of Educational Technology.

[CR10] Kirkwood A, Price L (2014). Technology-enhanced learning and teaching in higher education: What is ‘enhanced’and how do we know? A critical literature review. Learning, Media and Technology.

[CR11] Mayrberger, K., & Kumar, S. (2014). *Mediendidaktik und Educational Technology. Zwei Perspektiven auf die Gestaltung von Lernumgebungen mit digitalen Medien*. In Rummler, Klaus (Ed): Medien in der Wissenschaft, 67. Lernräume gestalten - Bildungskontexte vielfältig denken. Münster, Germany: Waxmann Verlag. 44–55.

[CR12] Moher D, Liberati A, Tetzlaff J, Altman DG (2009). Preferred reporting items for systematic reviews and meta-analyses: The PRISMA Statement. PLoS Medicine.

[CR13] Orey M, Branch RM (2017). Educational media and technology yearbook (Vol. 40).

[CR14] Pammer-Schindler V, Wild F, Fominykh M, Ley T, Perifanou M, Soule MV, Hernández-Leo D, Kalz M, Klamma R, Pedro L, Santos C, Glahn C, Economides AA, Parmaxi A, Prasolova-Førland E, Gillet D, Maillet K (2020). Interdisciplinary doctoral training in technology-enhanced learning in Europe. Frontiers in Education.

[CR15] Rousseeuw PJ (1987). Silhouettes: A graphical aid to the interpretation and validation of cluster analysis. Journal of Computational and Applied Mathematics.

[CR16] Scanlon, E., & Taylor, J. (2016). Is technology enhanced learning an interdisciplinary activity? In: *Proceedings of the 10th International Conference on Networked Learning 2016* (Cranmer, S.; Dohn, N. B.; de Laat, M.; Ryberg, T. and Sime, J. A. eds.), pp. 129–133.

[CR17] Shen CW, Ho JT (2020). Technology-enhanced learning in higher education: A bibliometric analysis with latent semantic approach. Computers in Human Behavior.

[CR18] Sommerhoff D, Szameitat A, Vogel F, Chernikova O, Loderer K, Fischer F (2018). What do we teach when we teach the learning sciences? A document analysis of 75 graduate programs. Journal of the Learning Sciences.

[CR19] Spector JM (2015). The changing nature of educational technology programs. Educational Technology.

[CR20] Tibshirani R, Walther G, Hastie T (2001). Estimating the number of clusters in a data set via the gap statistic. Journal of the Royal Statistical Society: Series B (statistical Methodology).

[CR21] Troelsen R, Zeuner L, Jensen A (2015). Interdisiplinære universitetsutdannelser. Hvordan sikres sammenhengskraften?. Uniped.

[CR22] Ward JH (1963). Hierarchical grouping to optimize an objective function. Journal of the American Statistical Association.

[CR23] Yang M (2009). Making interdisciplinary subjects relevant to students: An interdisciplinary approach. Teaching in Higher Education.

